# Supplementary material for: Dysregulated cholesterol homeostasis results in resistance to ferroptosis increasing tumorigenicity and metastasis in cancer
Source: Nat Commun. 2021 Aug 24;12:5103. doi: 10.1038/s41467-021-25354-4 (PMC8385107; doi:10.1038/s41467-021-25354-4)
Supplement: Supplementary file 1 — Supplementary Information [file 41467_2021_25354_MOESM1_ESM.pdf]

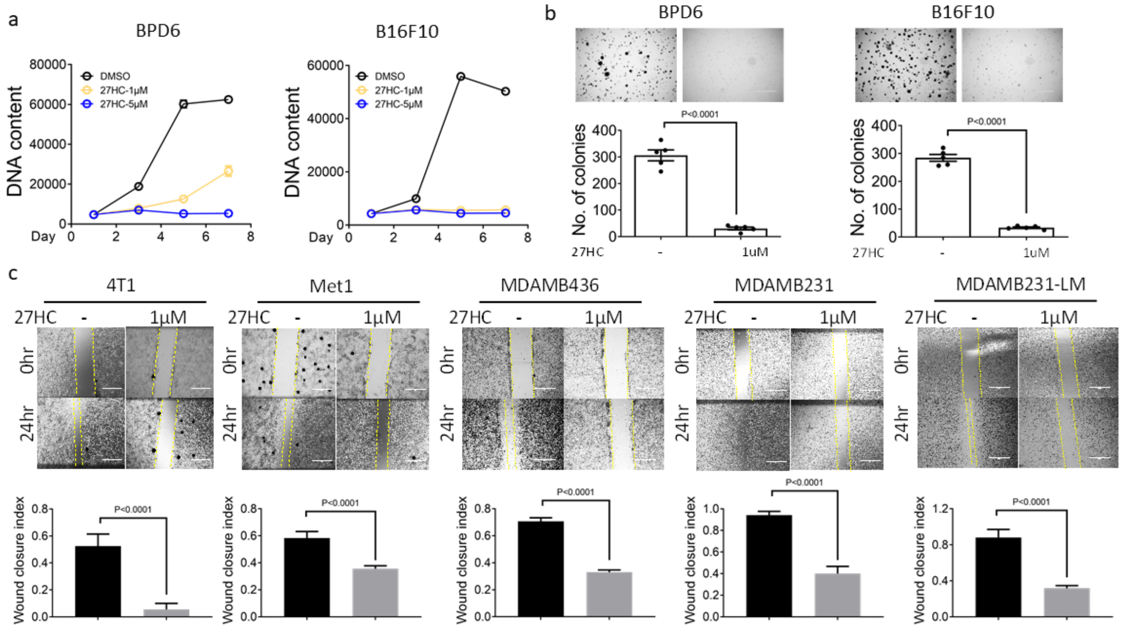

Supplementary Fig. 1. 27HC inhibits the growth and migration of ER-negative cancer cells. **a.** Time-dependent cell growth curves of ER-negative melanoma cancer cells, BPD6 (left) and B16F10 (right) treated with 0.1% DMSO or 27HC (1 and 5µM) in normal lipid serum-containing media. Cells were harvested at different time points and cell growth was assessed by measuring DNA content using Hoechst 33258. **b.** Soft agar assay showing that 27HC (1µM) inhibits the colony formation of BPD6 (left) and B16F10 (right) cells. **c.** ER-negative breast cancer cells 4T1, Met1, MDAMB436, MDAMB231 and MDAMB231-LM were treated with 0.1% DMSO or 27HC (1µM), and cell migration was determined by a scratch assay. Representative phase-contrast images of cancer cells at 0- and 24-hours after scratching are shown in upper panels. Data plotted as mean ± SEM from three technical replicates and are representative of results from two independent experiments. Data are plotted as mean ± SEM as representative results from two independent experiment; n=5 wells of cells (a and b); n=10-18 random field measurements from 3 trans-well chambers. (c). Two-sided unpaired Student's *t*-test (b and c). Numerical source data are reported in the Source Data File.

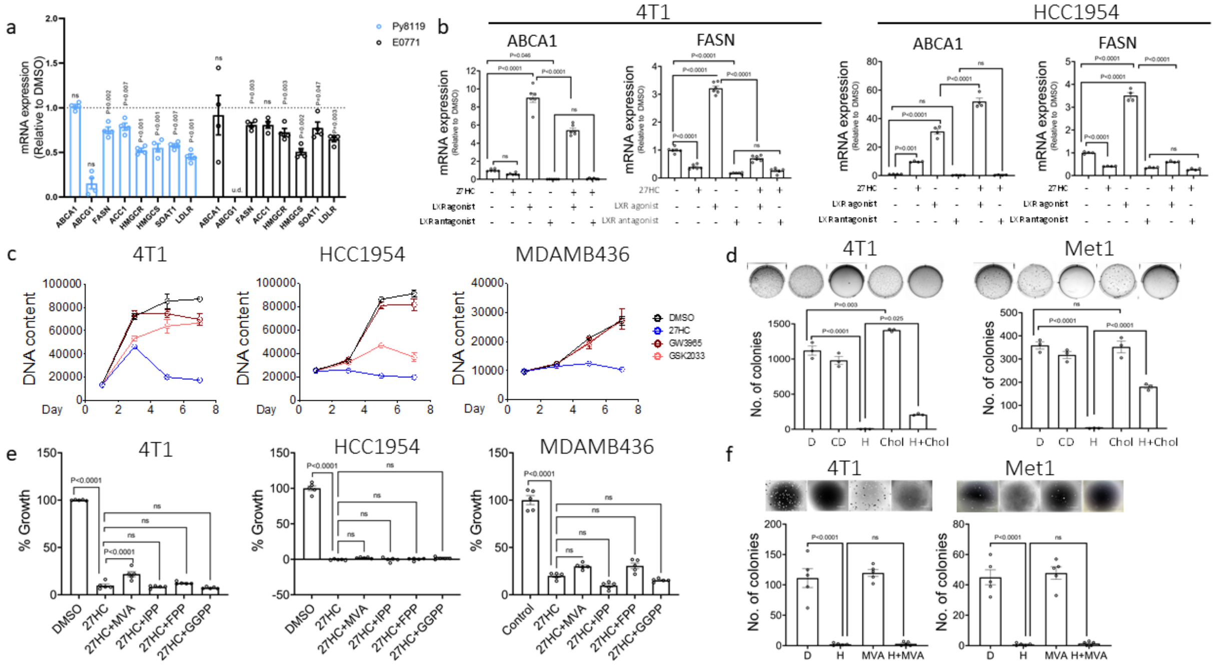

Supplementary Fig. 2. 27HC inhibits the growth of breast cancer cells via interfering with lipid metabolism. a. qRT-PCR analysis of the mRNA expression levels of LXR, SREBP1c and SREPB2 target genes involved in lipid metabolism from breast cancer cells, Py8119 and E0771, treated with 0.1% DMSO and 27HC (5µM) for 48 hours. Data are plotted as mean ± SEM, n=4 wells of cells. **b.** 4T1 and HCC1954 cells were treated with 27HC (5µM), LXR agonist (GW3965, 5µM) and antagonist (GSK2033, 5µM) for 48 hours. RNA was extracted followed by qRT-PCR to determine the expression of LXR target genes, ABCA1 and FASN. Data are plotted as mean ± SEM, n= 6 wells of cells (4T1) and 4 wells of cells (HCC1954). Results were also repeated in Met1 cells. **c.** 4T1, MDAMB436 and HCC1954 were treated with 27HC (5µM), LXR agonist (GW3965, 5µM) and antagonist (GSK2033, 5µM) for indicated time and cell growth was assessed by DNA content assay. Data are plotted as mean ± SEM as representative results from four independent experiments, n=5 wells of cells. **d.** 4T1 and Met1 cells growing in soft agar treated with the combination of 27HC (1µM) and cholesterol (10µM). Supplementation with cholesterol attenuated 27HC-dependent inhibition of colony formation. D-DMSO, CD-cyclodextrin, H-27HC, Chol-cholesterol. Data are plotted as mean ± SEM, n=3 wells of cells. **e.** Supplementation with upstream metabolites of cholesterol biosynthesis in mevalonate metabolism pathway including MVA (500µM), IPP (10µM), FPP (10µM) and GGPP (10µM) cannot rescue 27HC growth inhibition of 4T1, MDAMB436 and HCC1954 cells. Data are plotted as mean ± SEM as representative results from two independent experiments, n=5 wells of cells. **f.** 4T1 and Met1 cells growing in soft agar were treated with 27HC (1µM) with or without MVA (500µM). MVA supplementation does not rescue 27HC inhibition of colony formation. D-DMSO, H-27HC, MVA-mevalonate. Data are plotted as mean ± SEM as representative results from three independent experiments, n=5 wells of cells. Statistics were derived from two-sided unpaired Student's *t*-test (a) and one-way ANOVA with Tukey's post hoc test (b, d, e and f). Numerical source data are reported in the Supplementary Data 1 and Source Data File.

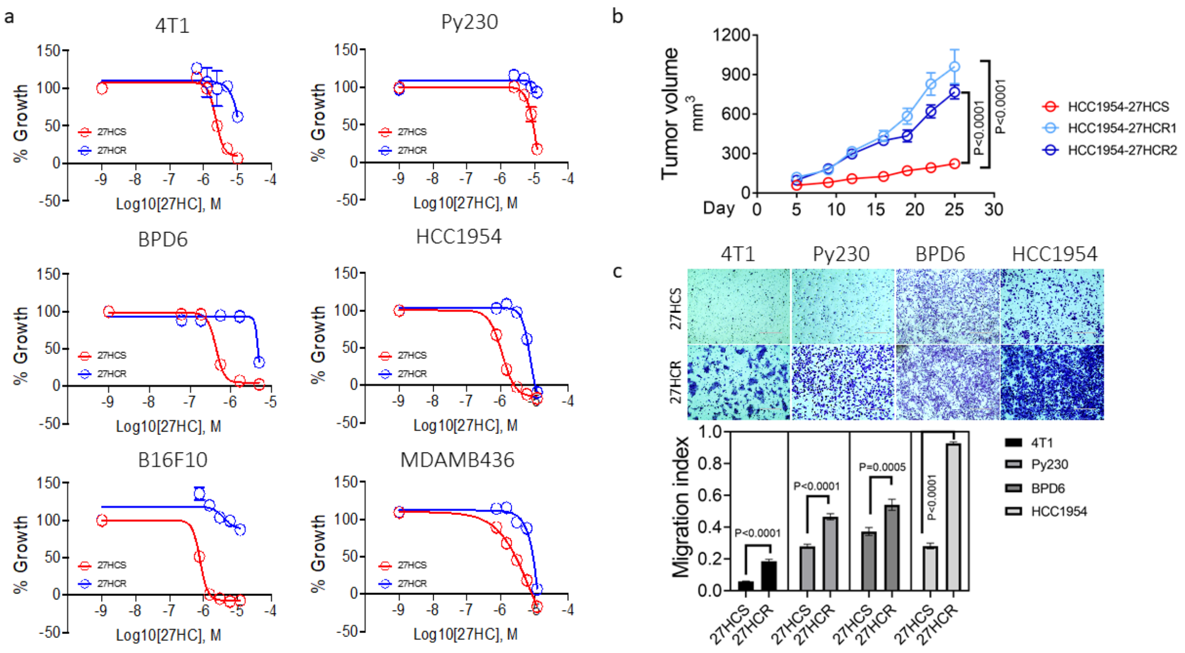

Supplementary Fig. 3. Chronic exposure to 27HC selects for cells with increased malignant phenotypes. a. Dose-response curves of 27HC treatment in both 27HCS and 27HCR derivative cells in multiple cancer models. Data are plotted as mean ± SEM as representative of results from three independent experiments. n=5 wells of cells. **b.** A total of $3X10^6$ cells in HCC1954-27HCS, -27HCR1 and -27HCR2 models were injected into the mammary fat pad of nude mice. Data are plotted as mean ± SEM; n= 10, 9 and 10 mice for HCC1954-27HCS, -27HCR1 and -27HCR2 cell lines, respectively; two-way ANOVA. **c.** Migratory capacity of 27HCS and 27HCR derivatives of 4T1, Py230, BPD6 and HCC1954 cells as determined using a trans-well migration assay. Representative pictures showing images of crystal violet staining. Migrated cells are plotted as mean ± SEM from n=15-21 random fields measurements from 3 trans-well chambers . Two-sided unpaired Student's *t*-test. Numerical source data are reported in the Source Data File.

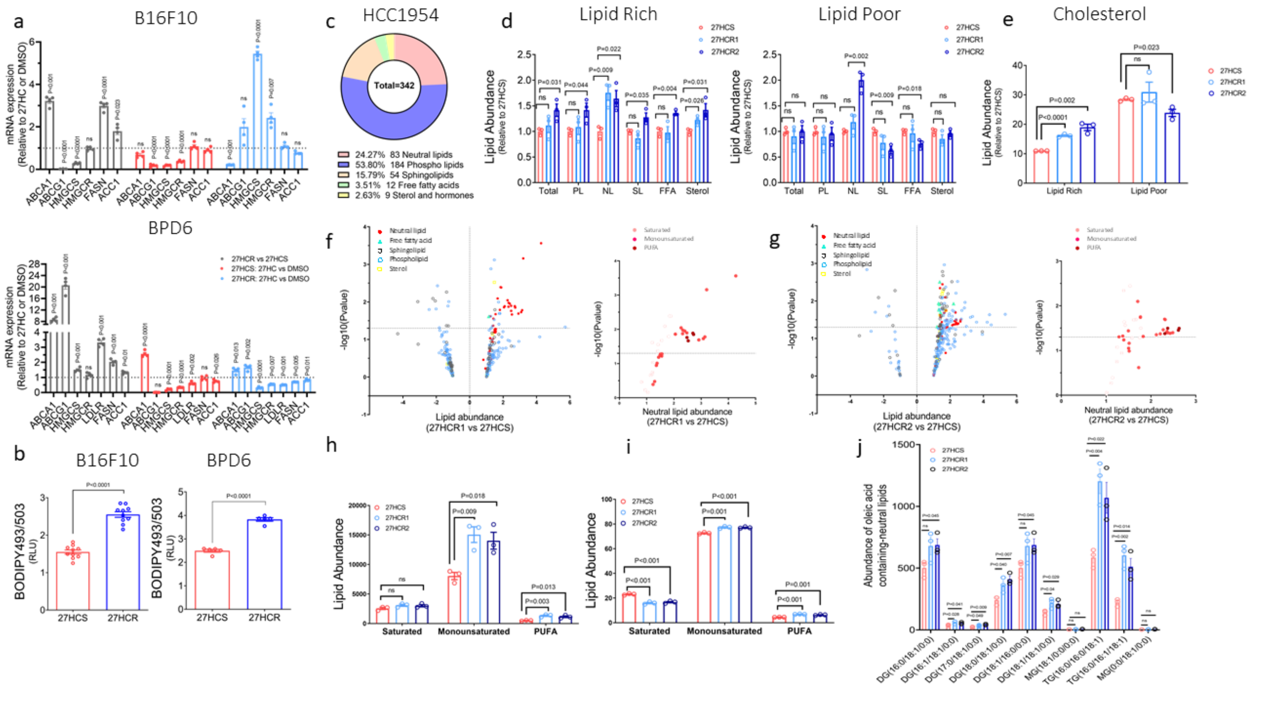

Supplementary Fig. 4. Dysregulation of lipid homeostasis in 27HCR cells. a. qRT-PCR analysis of the mRNA expression of lipid metabolism genes in 27HCS and 27HCR derivatives of B16F10 (upper) and BPD6 (lower) cells treated with DMSO (D, 0.1%) and 27HC (H, 5μM) for 24 hours. Data are plotted as mean ± SEM as representative results from two independent experiment; n=4 wells of cells; Two-sided unpaired Student's *t*-test. **b.** Lipid droplet content in 27HCS and 27HCR derivatives of B16F10 (left) and BPD6 (right) cells was evaluated by BODIPY493/503 staining. Data are plotted as mean ± SEM; n=10 wells of cells (B16F10) and 5 wells of cells (BPD6). Two-sided unpaired Student's *t*-test. **c.** Lipid composition of HCC1954 cells showed that phospholipid is the most abundant lipid. **d.** HCC1954-27HCS and -27HCR cells were cultured in lipid rich (FBS) and lipid poor (CFS) conditions, and relative abundance of total, PL (phospholipid), NL (neutral lipid), SL (sphingolipid), FFA (free fatty acid) and sterols were compared between 27HCS and 27HCR cells. **e.** Total cholesterol content in HCC1954-27HCS and -27HCR cells grown in lipid rich and lipid poor conditions. **f-g.** Volcano plots with fold change and p value of total lipid species (left) and neutral lipid species (right) in 27HCR1 (f) and 27HCR2 (g) compared with 27HCS in HCC1954 cells. **h-i.** Relative abundance (h) of saturated, monounsaturated and polyunsaturated fatty acid containing neutral lipid were compared between 27HCS and 27HCR cells grown in lipid rich condition. Percentage of saturated, monounsaturated and polyunsaturated fatty acid containing neutral lipid in total neutral lipid was also compared between 27HCS and 27HCR cells and plots are shown (i). **j.** Relative abundance of oleic acid containing neutral lipids were compared between 27HCS and 27HCR cells grown in lipid rich condition. Data plotted as mean ± SEM; n= 3 wells of cells (d-i). One-way ANOVA with Tukey's post-hoc test (d, e, h, i and j). Numerical source data are reported in the Supplementary Data 4 and Source Data File.

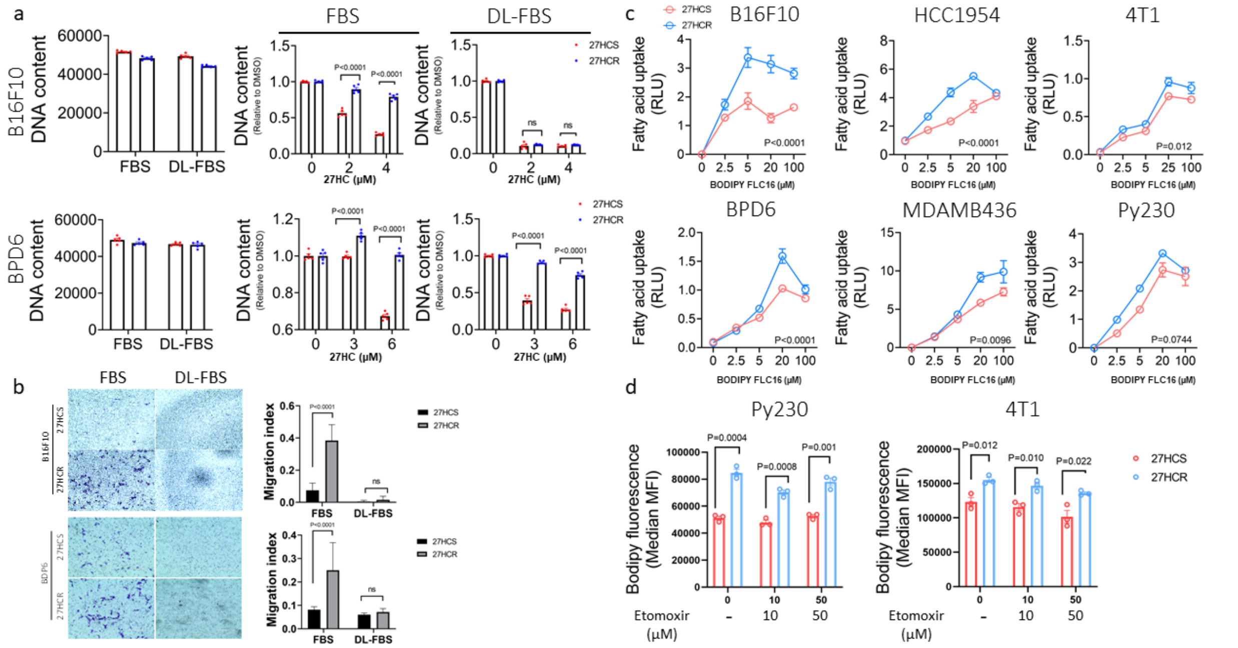

Supplementary Fig. 5. Increased lipid accumulation is characteristic of cells that are resistant to the antiproliferative activities of 27HC. **a.** 27HCS and 27HCR derivatives of B16F10 (upper panel) and BPD6 (lower panel) were cultured in lipid rich (FBS) and lipid depleted (DL-FBS) serum-containing media (left panels) and treated with 0.1% DMSO or 27HC at indicated doses for 72 hours (middle and right panels). Cell growth was assessed by measuring DNA content as a surrogate. Data are plotted as mean ± SEM from five technical replicates. One-way ANOVA with Tukey's post-hoc test. **b.** Migration capability of 27HCS and 27HCR B16F10 and BPD6 cells was determined using a trans-well migration assay in both lipid rich (FBS) and lipid depleted (DL-FBS) serum-containing media. Migrated cells are plotted as mean ± SEM from n=6-17 random fields measurements from 3 trans-well chambers ; One-way ANOVA with Tukey's post-hoc test. **c.** Lipid uptake in 27HCS and 27HCR cells in multiple cancer cells was assessed using a BODIPY FL-C16 staining assay. Data are plotted as mean ± SEM from five technical replicates and are representative of results from three independent experiments; two-way ANOVA. **d.** 27HCS and 27HCR derivatives of Py230 (left) and 4T1 (right) cells were cultured with or without etomoxir (10 and 50 µM) treatment. Lipid droplet content were visualized using BODIPY493/503 staining. Data are plotted as mean ± SEM from three technical replicates. One-way ANOVA with Tukey's post hoc test. Numerical source data are reported in the Source Data File.

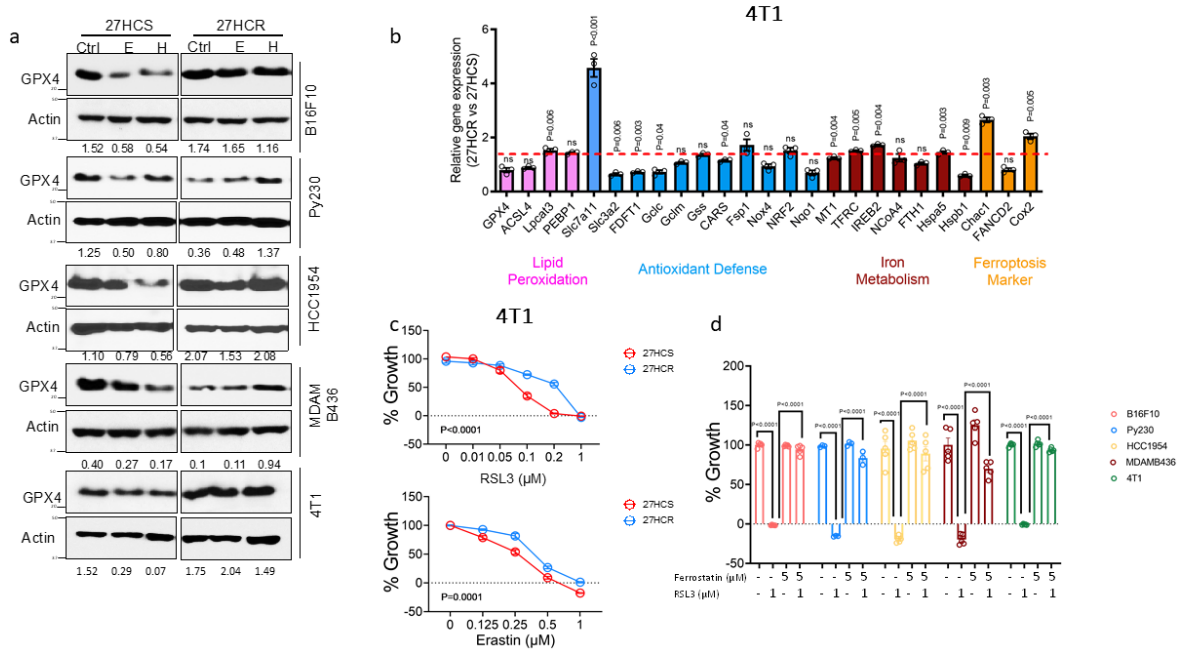

Supplementary Fig. 6. 27HCR cells are resistant to GPX4 inhibition induced ferroptosis. **a.** Western immunoblot analysis for the expression of GPX4 in 27HCS and 27HCR derivatives of B16F10, Py230, HCC1954, MDAMB436 and 4T1cells treated with system x_c^- inhibitor, erastin (E, 2.5 and 5μM) and 27HC (H, 5 and 8uM) for 24 or 48 hours. Representative results from two independent experiments. **b.** qRT-PCR profiling of genes, involved in the ferroptosis modulating signaling pathway, in 4T1-27HCS and -27HCR cells. Data plotted as mean ± SEM, n=3 wells of cells. Two-sided unpaired Student's *t*-test . **c.** Cell growth was evaluated in 4T1-27HCS and -27HCR cells treated with ferroptosis inducers including GPX4 inhibitors, RSL3 (left) and system x_c^- inhibitor, erastin (right) for 48 hours. Data plotted as mean ± SEM, n=5 wells of cells. Two-sided unpaired Student's *t*-test. **d.** RSL3 induced ferroptosis can be rescued with ferrostatin (5μM) in multiple cancer cell lines. Data plotted as mean ± SEM, n=3 wells of cells (Py230); n=5 wells of cells (B16F10, HCC1954, MDAMB436 and 4T1). One-way ANOVA with Tukey's post-hoc test. Unprocessed immunoblots and numerical source data are reported in the Supplementary Data 5 and Source Data File.

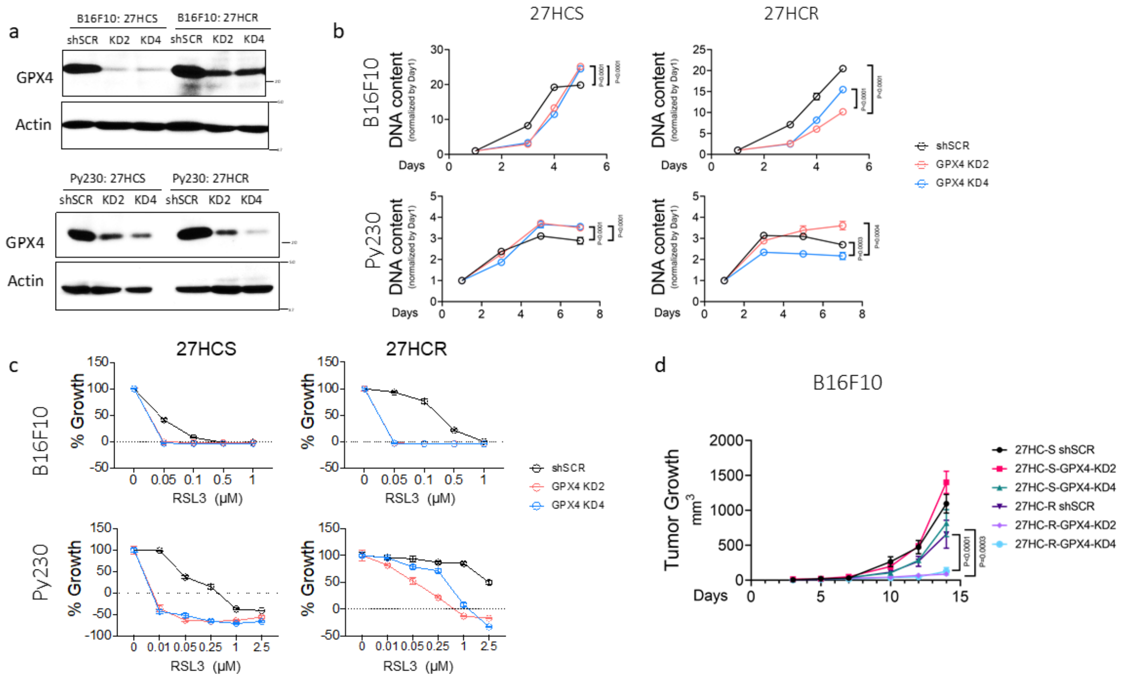

Supplementary Fig. 7. Inhibition of GPX4 sensitizes cancer cells to ferroptosis. a. Western immunoblot analysis of the expression of GPX4 and β-actin in GPX4 wild-type (shSCR) and knockdown (KD2 and KD4) in 27HCS and 27HCR derivatives of B16F10 cells (upper) and Py230 (lower) models. n= 1 well of cell; representative results from three independent experiments. **b.** Cell growth of the GPX4 wild-type (shSCR) and knockdown (KD2 and KD4) derivatives of B16F10- (upper) and Py230- (lower) 27HCS and 27HCR cells maintained in ferrostatin-free media. Data plotted as mean ± SEM, n=5 wells of cells. Two-way ANOVA. **c.** Cell growth analysis of GPX4 wild-type (shSCR) and knockdown (KD2 and KD4) derivatives of the B16F10- (upper) and Py230- (lower) 27HCS and 27HCR models following treatment with RSL3 or ML210 at the indicated doses. Data plotted as mean ± SEM representative of results from three (Py230) and four (B16F10) independent experiments; n=5 wells of cells. **d.** GPX4 wild-type (shSCR) and knockdown (KD2 and KD4) derivatives of the B16F10-27HCS and -27HCR cells (1X10^5) were orthotopically injected in nude mice (n=5 mice). Data plotted are mean ± SEM, two-way ANOVA. Unprocessed immunoblots and numerical source data are reported in the Source Data File.
