# Supplementary material for: Dysregulated cholesterol homeostasis results in resistance to ferroptosis increasing tumorigenicity and metastasis in cancer
Source: Nat Commun. 2021 Aug 24;12:5103. doi: 10.1038/s41467-021-25354-4 (PMC8385107; doi:10.1038/s41467-021-25354-4)
Supplement: Supplementary file 3 — Description of Additional Supplementary Files [file 41467_2021_25354_MOESM3_ESM.pdf]

Description of Additional Supplementary Files

Supplementary Data-1 qRT-PCR analysis of the mRNA expression levels of LXR, SREBP1c and SREBP2 target genes involved in lipid metabolism from 4T1, Met1, MDAMB436, HCC1954 and Py230 cells treated with 27HC (5 µM) for 24 -72 hours. Numerical source data to support Figure 1e and Supplementary Figure 2a.

Supplementary Data-2 qRT-PCR profiling of the expression of select genes involved in lipid metabolism in 27HCS- and 27HCR derivatives of 4T1, Py230, HCC1954, B16F10 and B6PD cells treated with 0.1% DMSO or 27HC (5 µM) for 24-72hr. Numerical source data to support Figure 3a and Supplementary Figure 4a.

Supplementary Data-3 The abundance of neutral lipids and phospholipid in 27HCS- and 27HCR-HCC1954 cells. Numerical source data to support Supplementary Figure 4c-j.

Supplementary Data-4 qRT-PCR analysis of the mRNA expression levels of genes involved in lipid uptake and trafficking in 27HCS- and 27HCR derivatives of 4T1, Py230, HCC1954 and B16F10 cell lines. Numerical source data to support Figure 3e.

Supplementary Data-5 qRT-PCR analysis of the expression of genes involved in ferroptosis pathway in 27HCS- and 27HCR derivatives of B16F10, Py230, HCC1954, MDAMB436 and 4T1 cells. Numerical source data to support Figure 4C and Supplementary Figure 6b.

Supplementary Data-6 names and sequences of qRT-PCR primers.
