# Supplementary material for: Dysregulated cholesterol homeostasis results in resistance to ferroptosis increasing tumorigenicity and metastasis in cancer
Source: Nat Commun. 2021 Aug 24;12:5103. doi: 10.1038/s41467-021-25354-4 (PMC8385107; doi:10.1038/s41467-021-25354-4)
Supplement: Supplementary file 10 — Reporting summary [file 41467_2021_25354_MOESM10_ESM.pdf]

nature research

Corresponding author(s): Donald P. McDonnell

Last updated by author(s): Jul 15, 2021

Reporting Summary

Nature Research wishes to improve the reproducibility of the work that we publish. This form provides structure for consistency and transparency in reporting. For further information on Nature Research policies, see our Editorial Policies and the Editorial Policy Checklist.

Statistics

For all statistical analyses, confirm that the following items are present in the figure legend, table legend, main text, or Methods section.

n/a	Confirmed	
☐	☒	The exact sample size (n) for each experimental group/condition, given as a discrete number and unit of measurement
☐	☒	A statement on whether measurements were taken from distinct samples or whether the same sample was measured repeatedly
☐	☒	The statistical test(s) used AND whether they are one- or two-sided<br>*Only common tests should be described solely by name; describe more complex techniques in the Methods section.*
☒	☐	A description of all covariates tested
☐	☒	A description of any assumptions or corrections, such as tests of normality and adjustment for multiple comparisons
☐	☒	A full description of the statistical parameters including central tendency (e.g. means) or other basic estimates (e.g. regression coefficient) AND variation (e.g. standard deviation) or associated estimates of uncertainty (e.g. confidence intervals)
☐	☒	For null hypothesis testing, the test statistic (e.g. F, t, r) with confidence intervals, effect sizes, degrees of freedom and P value noted<br>*Give P values as exact values whenever suitable.*
☒	☐	For Bayesian analysis, information on the choice of priors and Markov chain Monte Carlo settings
☒	☐	For hierarchical and complex designs, identification of the appropriate level for tests and full reporting of outcomes
☒	☐	Estimates of effect sizes (e.g. Cohen's d, Pearson's r), indicating how they were calculated

Our web collection on statistics for biologists contains articles on many of the points above.

Software and code

Policy information about availability of computer code

Data collection	no software was used.
Data analysis	Graphpad Prism version 6,7,8, 9 were used for statistical analysis; Image J 1.52K were used for migration assay quantitative analysis.

For manuscripts utilizing custom algorithms or software that are central to the research but not yet described in published literature, software must be made available to editors and reviewers. We strongly encourage code deposition in a community repository (e.g. GitHub). See the Nature Research guidelines for submitting code & software for further information.

Data

Policy information about availability of data

All manuscripts must include a data availability statement. This statement should provide the following information, where applicable:

- Accession codes, unique identifiers, or web links for publicly available datasets
- A list of figures that have associated raw data
- A description of any restrictions on data availability

All data supporting the findings of this study are available in the article and supplementary material, and can be provided upon reasonable request from the corresponding author (DPM).

Field-specific reporting

Life sciences study design

All studies must disclose on these points even when the disclosure is negative.

Sample size	Samples sizes were determined based on minimum number of replicate to achieve statistical power. n=3-5 were chosen for in vitro studies to test statistical significance, and n=5-10 were used for in vivo studies.
Data exclusions	No data were excluded; however, mice sometimes died during injection or experiments for experimental metastasis assay due to the growth of metastatic tumors, and were excluded from the end-point analysis of metastatic burden.
Replication	The number of replicates performed for each experiments were indicated in the figure legends. Data presented are either representative of replicates experiments of similar finding or average of replicates as indicated.
Randomization	Mice were randomized at Day 0 of tumor inoculation for all animal experiments. For in vitro experiment, cells were allocated into experimental groups based on its genetical alteration (tissue origins, 27HCS or 27HCR, GPX4 wt or KD); within the same cellular context, randomization was not required because we do not compare any treatment groups requiring randomization to form groups.
Blinding	The investigator and personnel were not blinded during this study in order to allow the investigators to a correct identification of samples and to ensure then a correct data collection.

Reporting for specific materials, systems and methods

We require information from authors about some types of materials, experimental systems and methods used in many studies. Here, indicate whether each material, system or method listed is relevant to your study. If you are not sure if a list item applies to your research, read the appropriate section before selecting a response.

Materials & experimental systems

n/a	Involved in the study	
☐	☒	Antibodies
☐	☒	Eukaryotic cell lines
☒	☐	Palaeontology and archaeology
☐	☒	Animals and other organisms
☒	☐	Human research participants
☒	☐	Clinical data
☒	☐	Dual use research of concern

Methods

n/a	Involved in the study	
☒	☐	ChIP-seq
☒	☐	Flow cytometry
☒	☐	MRI-based neuroimaging

Antibodies

Antibodies used	GPX4 Rabbit polyclonal antibody (ab41787, 1:1000, Abcam), Actin Mouse monoclonal antibody (A5441, 1:20,000, Sigma), HRP-conjugated secondary antibodies (1:5,000, BioRad #1706516 (Anti-Mouse IgG); #1706515 (Anti-Rabbit IgG).
Validation	Antibodies were validated according to the statement reported on the manufacturer's websites and published references. GPX4 antibody (https://www.abcam.com/glutathione-peroxidase-4-antibody-ab41787.html) and Actin antibody (https://www.sigmaaldrich.com/US/en/product/SIGMA/A5441?gclid=EAIaIQobChMIgd3LluDm8QIV9yCtBh0czgCAEAAYASAAEgKrZfD_BwE) GPX4 antibody was also validated in GPX4 KO cell lines (Supplementary Fig.7a)

Eukaryotic cell lines

Policy information about cell lines

Cell line source(s)	Human breast cancer cell lines (MCF7, MDAMB436, MDAMB231, MDAMB231-LM, HCC1954), mouse breast cancer cell lines (4T1, Met1, E0771 Py230, Py8119), mouse melanoma cell lines (BPD6 and B16F10) and human embryonic kidney cells 293FT. HCC1954, MDAMB231 and MDAMB231-LM cell lines were kindly provided by Dr. Joan Massague (Memorial Sloan Kettering Cancer Center). Py230 and Py8119 cell lines were kindly provided by Dr. Lesley Ellies (University of California- San Diego). BPD6 and B16F10 were kindly provided by Dr. Brent Hanks (Duke University), 293FT cells were kindly provided by Dr. Kris Wood (Duke University). All other cancer cell lines were obtained from American Type Culture Collection (ATCC).
Authentication	None of the cell lines used were authenticated in our lab, however, they were obtained directly from the deriving investigators or ATCC.
Mycoplasma contamination	All cell lines used were negative for mycoplasm
Commonly misidentified lines (See ICLAC register)	None

Animals and other organisms

Policy information about studies involving animals; ARRIVE guidelines recommended for reporting animal research

Laboratory animals	8-week-old female Balb/C, or C57BL/6, or athymic nude mice were used for xenograft and metastasis studies for 4T1, Py230 and GEMM6, HCC1954 cells, respectively. C57BL/6 (Stock No: 000664) and Balb/C (Stock No: 000651) mice were purchased from Jackson Laboratories and outbred nude mice were obtained from the Cancer Center Isolation Facility (Duke Cancer Institute, Durham, NC).
Wild animals	None
Field-collected samples	None
Ethics oversight	All of the studies involving the use of animals were conducted after prior approval by the Duke University Institutional Animal Care and Use Committee (IACUC).

Note that full information on the approval of the study protocol must also be provided in the manuscript.
